# AWRK6, a Novel GLP-1 Receptor Agonist, Attenuates Diabetes by Stimulating Insulin Secretion

**DOI:** 10.3390/ijms19103053

**Published:** 2018-10-07

**Authors:** Qiuyu Wang, Chunlin Zhao, Lili Jin, Hanyu Zhang, Qifan Miao, Hongsheng Liu, Dianbao Zhang

**Affiliations:** 1 School of Life Science, Liaoning University, Shenyang 110036, China; qiuyuwang@lnu.edu.cn (Q.W.); chunlinzhao.lnu@gmail.com (C.Z.); lilijin@lnu.edu.cn (L.J.); zhanghanyu.lnu@gmail.com (H.Z.); westg123456@gmail.com (Q.M.); 2 Research Center for Computer Simulating and Information Processing of Bio-macromolecules of Liaoning Province, Liaoning University, Shenyang 110036, China; liuhongsheng@lnu.edu.cn; 3 Department of Stem Cells and Regenerative Medicine, Key Laboratory of Cell Biology, National Health Commission of China, and Key Laboratory of Medical Cell Biology, Ministry of Education of China, China Medical University, Shenyang 110122, China

**Keywords:** AWRK6, antimicrobial peptide, GLP-1 receptor, agonist, diabetes, insulin

## Abstract

Diabetes is a metabolic disorder leading to many complications. The treatment of diabetes mainly depends on hypoglycemic drugs, often with side effects, which drive us to develop novel agents. AWRK6 was a peptide developed from the antimicrobial peptide Dybowskin-2CDYa in our previous study, and the availability of AWRK6 on diabetes intervention was unknown. Here, in vivo and in vitro experiments were carried out to investigate the effects of AWRK6 against diabetes. In diabetic mice, induced by high-fat diet followed by streptozocin (STZ) administration, the daily administration of AWRK6 presented acute and sustained hypoglycemic effects. The plasma insulin was significantly elevated by AWRK6 during an oral glucose tolerance test (OGTT). The relative β cell mass in diabetic mice was increased by AWRK6 treatment. The body weight and food intake were remarkably reduced by AWRK6 administration. In the mouse pancreatic β cell line Min6 cells, the intracellular calcium concentration was found to be enhanced under the treatment with AWRK6, and protein kinase A (PKA) inhibitor H-89 and Epac2 inhibitor HJC0350 represented inhibitory effects of the insulinotropic function of AWRK6. By FITC-AWRK6 incubation and GLP-1 receptor (GLP-1R) knockdown, AWRK6 proved to be a novel GLP-1R agonist. In addition, AWRK6 showed no toxicity in cell viability and membrane integrity in Min6 cells, and no hypoglycemia risk and no lethal toxicity in mice. In summary, AWRK6 was found as a novel agonist of GLP-1R, which could stimulate insulin secretion to regulate blood glucose and energy metabolism, via cAMP-calcium signaling pathway, without significant toxicity. The peptide AWRK6 might become a novel candidate for diabetes treatment.

## 1. Introduction

Diabetes mellitus (DM, commonly called diabetes) is a metabolic disorder with enhanced blood sugar, leading to many complications like cardiovascular disease, kidney disease, foot ulcers, and eye damage [1,2]. The incidence of diabetes in adults worldwide is as high as 8.3% and it would continue to rise [3]. Ninety percent of diabetes is type 2 diabetes, generally with obesity, deficiency in insulin secretion, and insulin resistance [4]. The treatment of diabetes mainly depends on hypoglycemic drugs such as insulin, sulfonylurea, metformin, α-glucosidase inhibitor, and thiazolidinediones, by increasing the insulin level, enhancing the insulin sensitivity, or decreasing the glucose absorption [5,6]. These drugs frequently present side effects such as weight gain, hypoglycemia, heart failure, and gastrointestinal problems [6,7], which drives us to develop novel agents.

GLP-1 receptor (GLP-1R), a G protein-coupled receptor expressed in various tissues such as islets, liver, and the nervous system, is involved in the control of blood sugar by binding GLP-1 and glucagon [8]. Thus, GLP-1R agonists has been considered as potential agents against diabetes. Several peptides were Food and Drug Administration (FDA)-approved, including an acylated GLP-1 named liraglutide, a GLP-1 analog exendin-4 found in Gila monster, and a sequence-modified exendin-4 named lixisenatide, while the risks of pancreatic, renal, and immune diseases limits their clinical application [9,10,11,12]. In previous studies, we have developed a peptide named AWRK6 (SWVGKHGKKFGLKKHKKH) according to the antimicrobial peptide Dybowskin-2CDYa, and AWRK6 was found to be effective in antimicrobial and anti-LPS-induced immune responses [13,14,15]. But the availability of AWRK6 on diabetes intervention is unknown.

In this study, we aimed to investigate the protective effects of AWRK6 against diabetes in mice, as well as the underlying mechanisms, which might offer a novel strategy for diabetes therapy.

## 2. Results

### 2.1. AWRK6 Reduced Blood Glucose in Diabetic Mice

**Figure 1:**
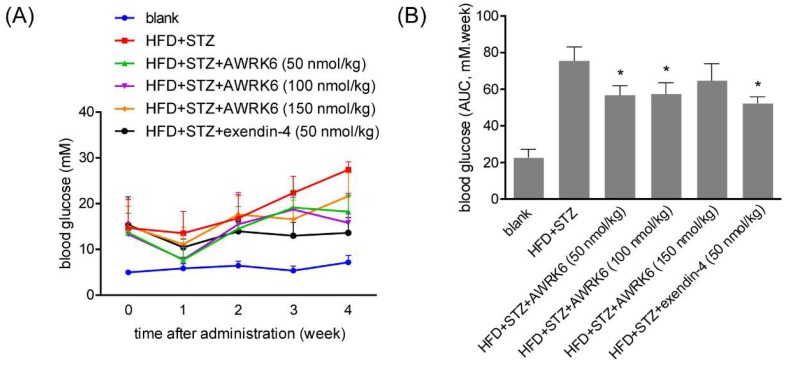

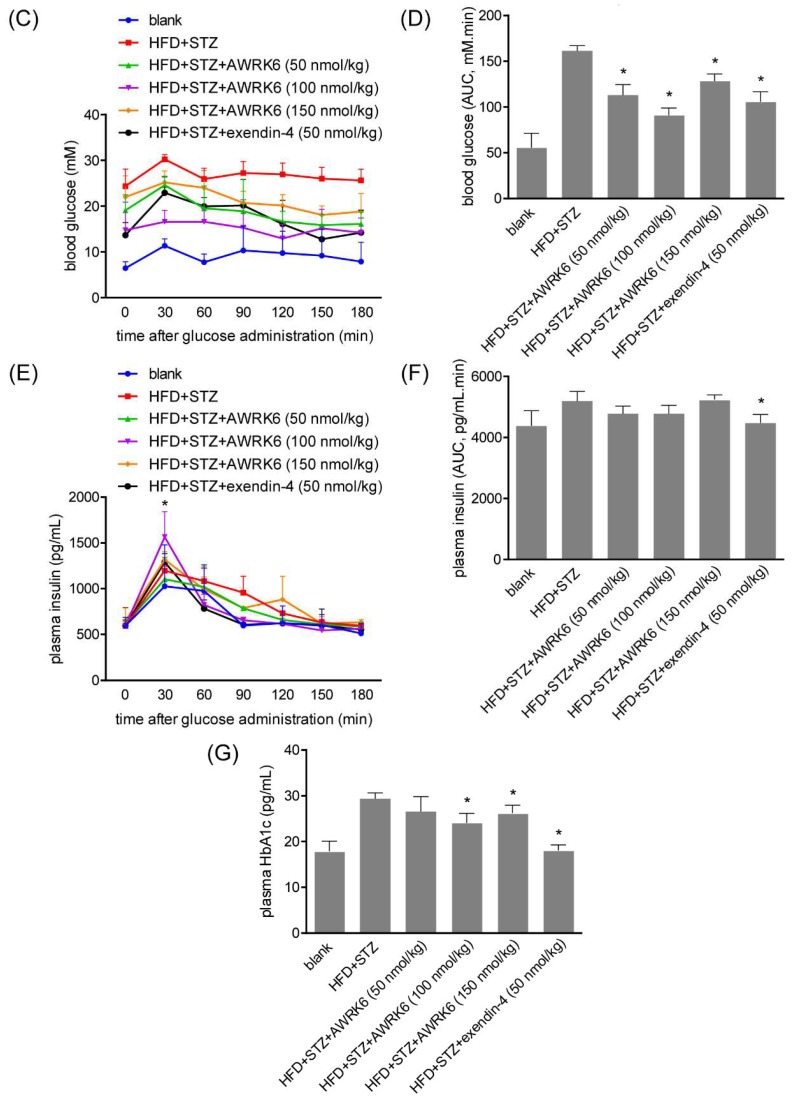
AWRK6 reduced blood glucose in diabetic mice. (**A**) The fasting blood glucose (FBG) in diabetic mice (high-fat diet (HFD) + streptozocin (STZ)) treated with AWRK6 during 4 weeks, detected by a glucometer. (**B**) The AUC (area under the curve) analysis of AWRK6 on fasting blood glucose of diabetic mice during 4 weeks. (**C**) The blood glucose in diabetic mice treated with AWRK6 for 4 weeks in an oral glucose tolerance test (OGTT), detected by a glucometer during 180 min. (**D**) The AUC analysis of the results of OGTT during 180 min. (**E**) The plasma insulin in diabetic mice treated with AWRK6 for 4 weeks in OGTT, detected by ELISA. (**F**) The AUC analysis of the results of plasma insulin in diabetic mice treated with AWRK6 for 4 weeks in OGTT. (**G**) The plasma HbA1c levels in diabetes mice after treatment with AWRK6 for 4 weeks, analyzed by ELISA. The error bar indicates standard deviation. * *p* < 0.05 compared with the diabetes groups.

To assess the anti-diabetic effects of AWRK6 in vivo, the diabetic mice were constructed using a high-fat diet (HFD) followed by streptozocin (STZ) administration. The daily administration of AWRK6 (50, 100, and 150 ng/kg) was carried out intraperitoneally over 4 weeks and the fasting blood glucose (FBG) was detected every 7 days. As shown in Figure 1A,B, the hypoglycemic effect of AWRK6 was evident during long-term treatment over 4 weeks, which was verified by the reduction of HbA1c (the β-*N*-1-deoxy fructosyl component of hemoglobin) levels (Figure 1G). Further, after the treatment for 4 weeks, an oral glucose tolerance test (OGTT) was performed. The increased blood glucose of diabetic mice was markedly reduced within 180 min, with comparable efficacy to exendin-4 (Figure 1C,D). Meanwhile, the plasma insulin was elevated significantly upon glucose administration for 30 min and decreased after 60 min, while the AUC (area under the curve) analysis presented no significant change (Figure 1E,F). These data indicated the efficacious acute and sustained hypoglycemic effects of AWRK6 in diabetic mice, possibly by inducing insulin secretion.

### 2.2. AWRK6 Increased β Cell Mass in Diabetic Mice

**Figure 2:**
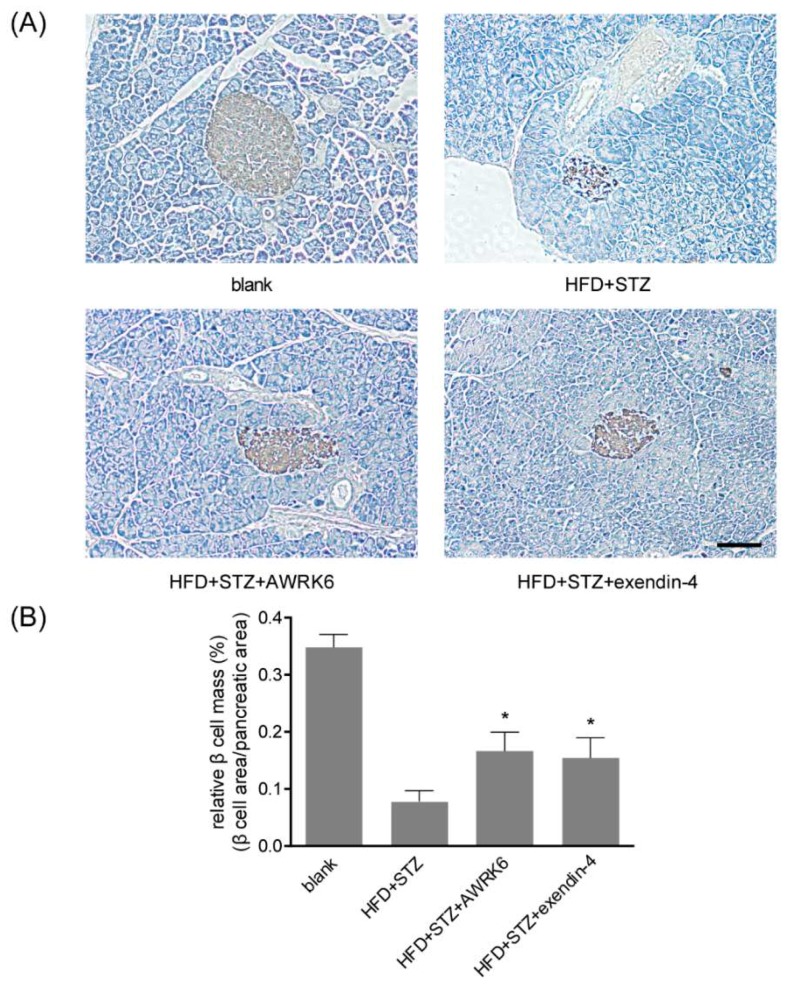
AWRK6 treatment increased the relative β cell mass in diabetic mice. (**A**) The representative immunohistochemistry (IHC) images of the pancreas, stained with an anti-insulin antibody. Bar indicates 100 μm. (**B**) The relative β cell mass was analyzed using ImageJ software. The error bar indicates standard deviation. * *p* < 0.05 compared with the diabetes groups.

In the diabetic mice model, STZ could cause β cell damage by triggering immune responses. To investigate the protective effects of AWRK6 against islet injury, the pancreas tissues of the mice were collected and fixed after the treatment with AWRK6 (100 nmol/kg) for 4 weeks. Paraffin sections were made and immunohistochemistry (IHC) analysis using an anti-insulin antibody was carried out. The morphology of pancreas was observed under a microscope and the relative β cell mass was analyzed using ImageJ software. As shown in Figure 2, the relative β cell mass was decreased by the treatment with HFD and STZ, to about 20% of the blank control. The AWRK6 treatment presented a significant increase of the relative β cell mass in the diabetic mice model, which was comparable with that of exendin-4. These results indicated that AWRK6 could repair islet damage in diabetic mice.

### 2.3. AWRK6 Decreased Food Intake and Body Weight

**Figure 3:**
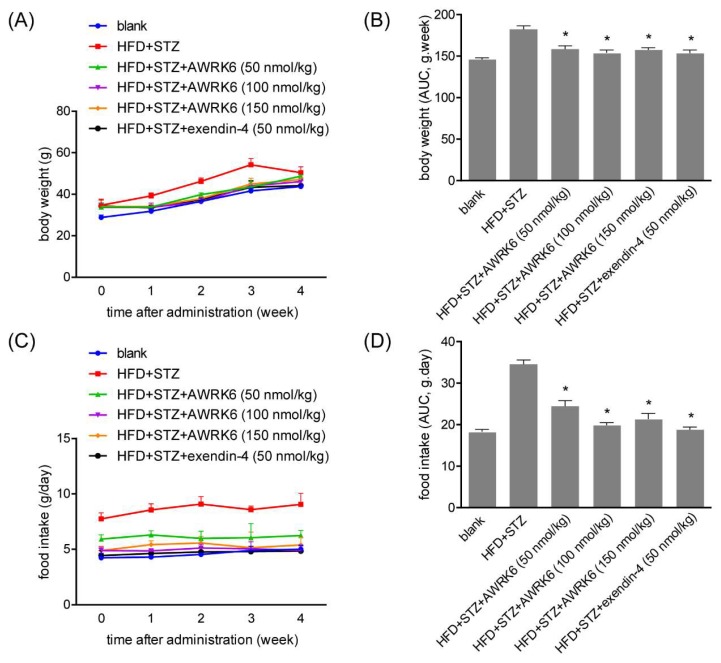
AWRK6 decreased food intake and body weight. (**A**) The body weight of diabetic mice treated with AWRK6 during 4 weeks. (**B**) The AUC analysis of AWRK6 on body weight of diabetic mice during 4 weeks. (**C**) The food intake of diabetic mice treated with AWRK6 during 4 weeks. (**D**) The AUC analysis of AWRK6 on food intake of diabetic mice during 4 weeks. The error bar indicates standard deviation. * *p* < 0.05 compared with the diabetes groups.

Considering that obesity is generally in close relationship with diabetes, the body weight and food intake were monitored during the treatment with AWRK6. For diabetic mice induced by high-fat feeding and STZ, body weight was significantly elevated and the daily treatment with AWRK6 over 4 weeks significantly decreased the body weight (Figure 3A,B). Further, the food intake also presented lower levels in the AWRK6-treated group, compared with the diabetes group (Figure 3C,D). Those suggested the positive role of AWRK6 in energy metabolism, which may involve the regulation of fat balance during energy utilization.

### 2.4. AWRK6 Induced Insulin in a cAMP-Dependent Manner

**Figure 4:**
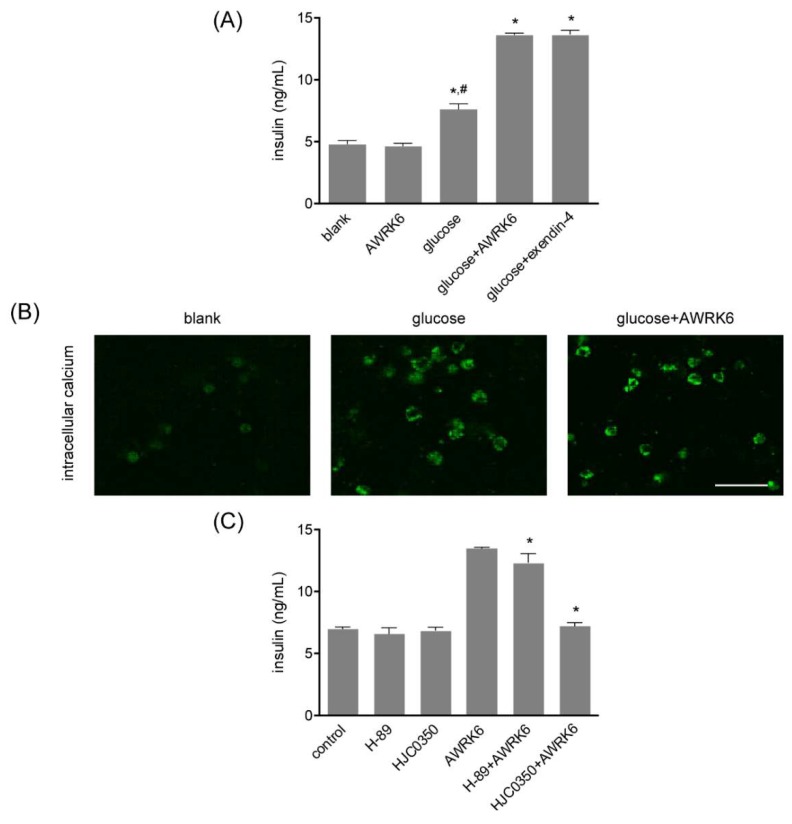
AWRK6 induced insulin in a cAMP-dependent manner. (**A**) The insulin secretion in Min6 cells treated with AWRK6, analyzed by ELISA. (**B**) The intracellular calcium in Min6 cells treated with AWRK6, determined using Fluo 3-AM. (**C**) The insulin secretion in Min6 cells treated with AWRK6, protein kinase A (PKA) inhibitor H-89, and Epac2 inhibitor HJC0350. * *p* < 0.05 compared with the glucose group for A, B, and compared with the AWRK6 group for C; ^#^ *p* < 0.05 compared with the blank group. The error bar indicates standard deviation. Scale bar indicates 100 μm.

To assess potential signaling bias, the mouse pancreatic β cell line Min6 cells were treated with AWRK6. The insulin in the culture medium was detected by ELISA, as shown in Figure 4A, and the insulin secretion was significantly elevated by the incubation with 50 nM AWRK6 and 25 mM glucose. AWRK6 presented no significant insulinotropic effect in Min6 cells without glucose, suggesting that AWRK6 could increase the insulin secretion under glucose response in β cells, with low risk for hypoglycemia under low glucose condition. By Fluo 3-AM, the intracellular calcium concentration in Min6 cells was found to be enhanced under the treatment with AWRK6; it was suggested that AWRK6 might induce the release of intracellular calcium pool to promote insulin secretion (Figure 4B). Further, protein kinase A (PKA) inhibitor H-89 and Epac2 inhibitor HJC0350 represented inhibitory effects of the insulinotropic function of AWRK6 (Figure 4C). Considering that cAMP exerts its effects via PKA and Epac2 by direct activation, these data proved that AWRK6 induced insulin secretion through cAMP-calcium signaling pathway under high glucose condition.

### 2.5. AWRK6 Was a GLP-1R Agonist

**Figure 5:**
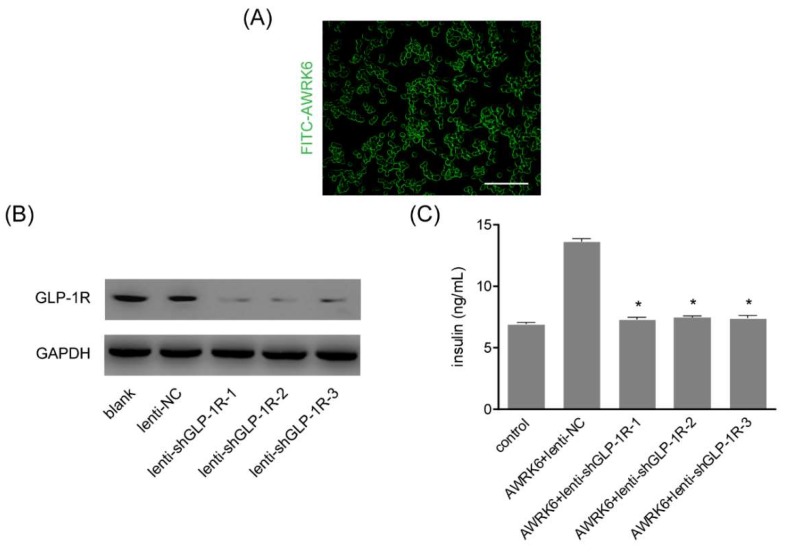
AWRK6 was a GLP-1 receptor (GLP-1R) agonist. (**A**) AWRK6 interacted with Min6 cells outer membrane, assayed by incubation with FITC-conjugated AWRK6. Scale bar indicates 100 μm. (**B**) The GLP-1R silencing using lentivirus was verified by western blotting. (**C**) The insulin secretion in GLP-1R-silenced Min6 cells treated with AWRK6. The error bar indicates standard deviation. * *p* < 0.05 compared with the AWRK6 + lenti-NC groups.

After incubating with FITC-conjugated AWRK6, the Min6 cells were found to be stained with fluorescent dye on the cell surface, suggesting that AWRK6 might interact with receptors on the cell membrane (Figure 5A). Then, GLP-1R was knockdown by using three lentiviruses and verified by western blotting (Figure 5B). The insulin in the culture medium was detected by ELISA, and it was found that GLP-1R silencing remarkably abated the insulinotropic effect of AWRK6 in Min6 cells (Figure 5C). Combined with the cAMP-calcium signaling involved in the insulinotropic AWRK6, AWRK6 was presumed to be a novel GLP-1R agonist.

### 2.6. AWRK6 Showed No Toxicity in Vitro and in Vivo

**Figure 6:**
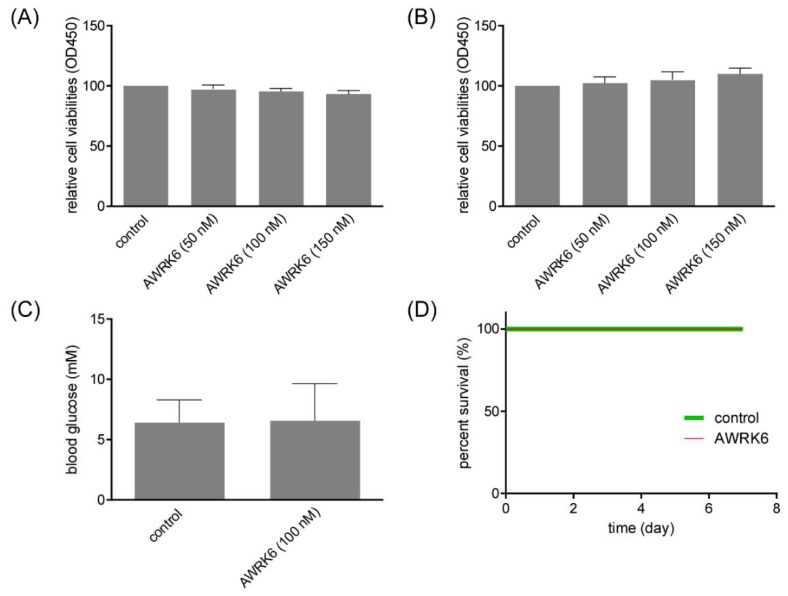
AWRK6 showed no toxicity in vivo and in vitro. (**A**) The cell viability of Min6 cells treated with AWRK6, analyzed by CCK-8 assay. (**B**) The lactate dehydrogenase (LDH) release of Min6 cells treated with AWRK6, analyzed by LDH assay. (**C**) The blood glucose in normal mice treated with AWRK6, analyzed using a glucometer. (**D**) The survival curve of diabetic mice treated with a high dose of AWRK6. The error bar indicates standard deviation.

For the potential application of AWRK6 against diabetes, it is essential to assess its toxicity in vitro and in vivo. The cell viability of Min6 cells treated with AWRK6 was determined by CCK-8 assay, and there was no significant reduction in the AWRK6-treated cells vs control group (Figure 6A). Considering that AWRK6 might interact with the cell membrane, the cell membrane integrity was analyzed using lactate dehydrogenase (LDH) assay. As shown in Figure 6B, the LDH release of Min6 cells presented no significant change upon the treatment of AWRK6. Further, the hypoglycemia risk of AWRK6 administration in normal mice was detected using a glucometer. AWRK6 showed negligible effect of AWRK6 on blood glucose in normal mice (Figure 6C). In addition, AWRK6 showed no lethal toxicity to diabetic mice (Figure 6D). These results provided positive support for the safety of AWRK6.

## 3. Discussion

With the increasing incidence of diabetes, strict glycemic control and treatment of complications are facing severe challenges. The strategies promoting insulin secretion have been the mainstay of the treatment of diabetes. In recent years, several peptides from frog skin, firstly identified by their antimicrobial or immunomodulatory activities, were proven to have the function of stimulating insulin release in vitro and in vivo, thus showing the potential of development into agents against diabetes [16]. In this study, we have investigated the protective effects of the novel peptide AWRK6, which was derived from the frog antimicrobial peptide Dybowskin-2CDYa, against diabetes in vivo and in vitro. In the diabetic mice constructed by a high-fat diet and STZ administration, after the treatment of AWRK6 by intraperitoneal injection, the plasma insulin was elevated and the blood glucose was reduced remarkably during short-term and long-term treatment, which was comparable with the positive control exendin-4. For the increasing impact of obesity on the incidence of diabetes, the demand for weight and blood glucose control is becoming more and more important [17]. Thus, the body weight and food intake of the diabetic mice were monitored during 28-day treatment with AWRK6, and it was shown that the body weight and food intake were reduced significantly vs control, indicating the regulation of AWRK6 on the balance during energy utilization. These results have proven the promising potential of AWRK6 as a novel agent against diabetes, partly through stimulating insulin secretion. Compared with the positive control exendin-4, AWRK6 showed no significant functional improvement, and while AWRK6 consisted of only 18 amino acids, its production cost was much lower than that of exendin-4 (39 amino acids). We will continue to study the potential therapeutic effects of AWRK6 on diabetes, including detailed mechanisms, bioavailability, and pharmacokinetics, meanwhile comparing it with more clinical drugs.

Generally, the secretion of insulin was regulated by the intracellular calcium concentration in β cells, which was under the control of ATP sensitive potassium channels, calcium channels, and intracellular calcium pools on the plasma membrane [18,19,20,21,22]. By the pretreatment with the calcium channel inhibitor verapamil and the extracellular calcium chelating agent EGTA in Min6 cells, the intracellular calcium pools proved to be the main source of the elevated intracellular calcium. The calcium elevation in the β cells could trigger insulin secretion and the signaling is generally considered to be amplified by cAMP, a universal second messenger of many G-protein-coupled receptors, and regulates a wide variety of cellular events [23]. Generally, cAMP exerts its effects via protein kinase A (PKA) and the guanine nucleotide exchange factor Epac [24,25]. Thus, the insulin secretion was determined under the treatment with PKA inhibitor and Epac2 inhibitor in Min6 cells, and PKA and Epac2 were found to be crucial for the elevation of insulin induced by AWRK6. Therefore, we concluded that AWRK6 stimulated insulin secretion in a cAMP-calcium-dependent manner. To further investigate the interaction of AWRK6 with β cells, GLP-1, a G-protein-coupled receptor involved in the control of blood sugar, was silenced by lentiviruses and the insulin secretion induced by AWRK6 was repressed. Accordingly, we luckily found that AWRK6 might be a novel agonist of GLP-1R. It is important to verify the direct interaction of AWRK6 with GLP-1R, while Co-IP and GST-pulldown are not available now, due to the lack of a specific antibody for the novel synthetic peptide AWRK6. More laboratory works are needed for investigating the detailed mechanisms.

In previous studies, GLP-1R agonists such as GLP-1, Exenatide, and Liraglutide, present multiple biological actions in type 2 diabetes, including restoration glucose-dependent stimulation of insulin secretion, more adequate insulin secretory response after meals, suppression of glucagon secretion, and enhancement of glucagon secretion when plasma glucose is low [26]. Here, AWRK6 proved to reduce blood glucose as a novel GLP-1R agonist. AWRK6 is expected to have various beneficial biological effects like other GLP-1R agonists, especially for the effect of AWRK6 on glucagon secretion. Further studies are essential for more biological effects of AWRK6.

In this study, we have found the peptide AWRK6 as a novel agonist of GLP-1R, which could stimulate insulin secretion to regulate blood glucose and energy metabolism, via cAMP-calcium signaling pathway, with no significant toxicity. The peptide AWRK6 might become a novel candidate for diabetes treatment.

## 4. Materials and Methods

### 4.1. Peptides

The synthetic peptide AWRK6 and FITC-conjugated AWRK6 were obtained from GL Biochem Corporation (Shanghai, China), which were purified by RP-HPLC for a high purity of 99% [14]. Exendin-4 was purchased from BioVision (Milpitas, CA, USA). The lyophilized peptides were dissolved in physiological saline for the subsequent experiments.

### 4.2. Mice Model

Male Kunming mice (18–22 g) were obtained from Changsheng Biotechnology (Shenyang, China) and housed in a temperature-controlled environment with free access to food and water. To generate diabetic model mice, the mice were fed a high-fat diet with 60% fat (Changsheng Biotechnology) for 3 weeks, then administrated with STZ (50 mg/kg, Sigma-Aldrich, Shanghai, China) by intraperitoneal injection daily for 5 days. After the high-fat diet followed by STZ administration, the mice were treated with AWRK6 through intraperitoneal injection once per day. Exendin-4 was used as a positive control and physiological saline was used as a blank control. The body weight and food intake were monitored weekly. Nine mice were used for each group. The animal experiments were approved by the Ethics Committee of Liaoning University (20150011, 23 February 2015) and conducted according to the ethical guidelines for the Care and Use of Laboratory Animals.

### 4.3. Blood Glucose, Insulin, and HbA1c Measurement

Blood samples were collected from a tail vein and the blood glucose was estimated using ACCU-CHEK glucometer (Roche, Shanghai, China). The insulin (in plasma and culture medium) and HbA1c were determined using ELISA kit according to the manufacturer’s instructions. The insulin kit was purchased from Anoric Bio-technology (Tianjin, China). The HbA1c kit was purchased from Jiancheng Bioengineering Institute (Nanjing, China). The absorbance at 450 nm was detected on iMARK microplate reader (Bio-Rad, Hercules, CA, USA).

### 4.4. Histopathological Examination

The pancreas tissues were collected and fixed with 10% formalin. Then the tissues were embedded in paraffin and 5 μm sections were made. The sections were stained with the rabbit anti-insulin antibody (Proteintech, Wuhan, China) and visualized using UltraSensitive SP IHC Kit (Maxin, Fuzhou, China) according to the manufacturer’s instructions. The sections were observed under an Olympus CK31 microscope and the images were analyzed using ImageJ software (NIH, Bethesda, MD, USA). For each group, 15 pancreatic histological slides (3 200 μm-apart sections for each sample) including at least 100 islets were analyzed. The relative β cell mass was calculated as the area of insulin-positive cells/total pancreatic area [27].

### 4.5. Cell Culture

The mouse pancreatic β cell line Min6 cells were obtained from Bogoo Biotech (Shanghai, China). The cells were cultured in DMEM (high glucose, Hyclone, Beijing, China) supplemented with 15% fetal bovine serum (Hyclone, Beijing, China), 0.5% 2-Mercaptoethanol (Gibco, Shanghai, China) and 1% penicillin streptomycin (Hyclone) at 37 °C in a humidified atmosphere containing 5% CO_2_. To induce insulin secretion, Min6 cells were cultured in serum-free medium for 2 h, washed and incubated in KRB (Krebs-Ringer bicarbonate buffer) with 2 mM glucose for 30 min. Then the cells were stimulated with glucose (25 and 30.6 mM), AWRK6 (50 nM), and exendin-4 (20 nM) in KRB for 4 h, and the subsequent experiments were carried out as described. To evaluate the involvement of cAMP pathway in AWRK6-induced insulin secretion, PKA inhibitor H-89 (48 nM, MedChem Express, Shanghai, China) and Epac2 inhibitor HJC0350 (0.3 μM, MedChem Express) were used for the pretreatment of Min6 cells at 30 min before glucose stimulation.

### 4.6. Intracellular Calcium Determination

The intracellular calcium of Min6 cells was determined by Fluo 3-AM (Dojindo, Shanghai, China). Briefly, Min6 cells were seeded in confocal dishes, pretreated with verapamil (50 μM, Solarbio), EGTA (5 μM, Solarbio) and stimulated with glucose as indicated. The cells were washed with KRB and incubated with Fluo 3-AM working solution (5 μM in KRB) for 45 min at 37 °C in the dark. The cells were washed again and incubated for 30 min, then observed and imaged under FV1000 confocal laser scanning microscope (Olympus, Tokyo, Japan).

### 4.7. GLP-1R Silencing by Lentivirus

GLP-1R Silencing was carried out using lentiviruses which were prepared by Micro Helix company (Beijing, China). Three shRNAs against mouse GLP-1R and scrambled control were used. The Min6 cells were seeded in 6-well plates and infected with these lentiviruses, the subsequent experiments were carried out at 48 h after infection.

### 4.8. Western Blotting

The membrane protein was prepared using Mem-PER Plus Membrane Protein Extraction Kit (Thermo Scientific, Shanghai, China). Equal amounts of protein were separated by SDS-PAGE and transferred to NC membranes. The membranes were incubated overnight at 4 °C with antibodies against mouse GLP-1R (1:2000, Proteintech, Wuhan, China), Epac2 (1:2000, Abcam, Shanghai, China) and GAPDH (1:10,000, ZSGB Bio, Beijing, China), followed by incubation with HRP-conjugated secondary antibodies (1:10,000, Abbkine Biotech, Wuhan, China). Protein bands were visualized by ECL Plus Reagent (Solarbio, Beijing, China) and imaged on MicroChemi chemiluminescence detection system (DNR Bio Imaging System, Neve Yamin, Israel). The protein bands were analyzed using ImageJ software (NIH, Bethesda, MD, USA).

### 4.9. Cell Viability Assay

The cell viability was assayed using CCK-8 (Dojindo). The cells were plated into 96-well plates and incubated overnight. After indicated treatments for 24 h, 10 μL CCK-8 reagent was added to each well and incubated for 2 h. The absorbance at 450 nm was detected on iMARK microplate reader (iMARK, Bio-Rad).

### 4.10. LDH Release Assay

Lactate dehydrogenase assay kit (Jiancheng Bioengineering Institute, Nanjing, China) was applied for LDH release analysis. The Min6 cells were seeded in 96-well plates and incubated overnight. The cells were treated as indicated for 24 h and the supernatant was transferred to another 96-well plates for detection according to the manufacturer’s instructions. The absorbance at 450 nm was detected using iMARK microplate reader.

### 4.11. Statistical Analysis

The survival rates between groups were analyzed using the Mantel-Cox Log-rank test. Other data from three replicas were presented as mean ± SD and analyzed by student’s *t* test or one-way ANOVA followed by multiple comparisons. *p* < 0.05 was considered statistically significant.
